# Expenses of the Brazilian Public Healthcare System with chronic kidney disease

**DOI:** 10.1590/2175-8239-JBN-3918

**Published:** 2018-06-04

**Authors:** Paulo Roberto Alcalde, Gianna Mastroianni Kirsztajn

**Affiliations:** 1 Universidade Federal de São Paulo, Division of Nephrology, São Paulo, SP, Brasil.

**Keywords:** Renal Insufficiency, Chronic, Health Care, Cost Control, Health Systems, Hospitalization, Database, Insuficiência Renal Crônica, Atenção à Saúde, Controle de Custos, Sistemas de Saúde, Hospitalização, Base de Dados

## Abstract

**Introduction:**

The prevalence of chronic kidney disease (CKD) is increasing worldwide, with costs that can be impeditive.

**Objective:**

To establish the expenses of the Brazilian Public Healthcare System (SUS), with hospitalizations due to CKD and related diseases; evaluating expenses with renal replacement therapy (RRT).

**Methods:**

We have assessed the values paid by the SUS in the triennium 2013-2015, for estimating annual expenses with CKD treatment and hospitalization, associated diseases, and exams.

**Results:**

There was an increase in hospitalization by all causes in Brazil during this triennium. CKD and associated diseases corresponded to 1.82% and 5.79% of hospitalizations due to all causes in Brazil, and 2.87% and 10.10% of all expenses, respectively. Kidney grafts from deceased donors corresponded to 76% of hospitalizations and 80% of expenses with transplantation. There was a decrease in transplantation from living donors. There was an increase in the number of exams of 11.94% and of 10.95% in the expenses. There was a decrease in the number of procedures and expenses in intermittent peritoneal dialysis (IPD) and related procedures; but other procedures increased. Hemodialysis (3 weekly sessions) corresponded to 95.96% of procedures and 96.07% of expenses with dialysis in general.

**Conclusion:**

Renal diseases and some of the main related diseases corresponded to 12.97% of the expenses in the triennium 2013-2015, and RRT to more than 5% of the SUS expenses with medium and high complexity healthcare. Such high expenses determine great concerns on the future maintenance of treatment for stage-5 CKD in Brazil and countries in similar or worse conditions of development.

## RESUMO

**Introdução:**

A prevalência de doença renal crônica (DRC) está aumentando em todo o mundo, com custos que podem ser impeditivos.

**Objetivo:**

Estabelecer os gastos do Sistema Único de Saúde (SUS) com internação hospitalar por DRC e doenças relacionadas; avaliar os gastos com terapia renal substitutiva (TRS).

**Métodos:**

Avaliamos os valores pagos pelo SUS no triênio 2013-2015, para estimar as despesas anuais com tratamento da DRC e internação por DRC, doenças associadas e exames.

**Resultados:**

Houve aumento da hospitalização por todas as causas no Brasil neste triênio. A DRC e as doenças associadas corresponderam a 1,82% e 5,79% das internações hospitalares por todas as causas no Brasil, e 2,87% e 10,10% de todas as despesas, respectivamente. Os enxertos renais com doadores falecidos corresponderam a 76% das internações e 80% das despesas com o transplante. Houve uma diminuição em transplantes de doadores vivos. Houve um aumento no número de exames de 11,94% e em despesas de 10,95%. Houve uma diminuição no número de procedimentos e despesas em diálise peritoneal intermitente (IPD) e procedimentos relacionados; mas outros procedimentos aumentaram. A hemodiálise (3 sessões semanais) correspondeu a 95,96% dos procedimentos e 96,07% dos gastos com diálise em geral.

**Conclusão:**

As doenças renais e algumas das principais doenças relacionadas corresponderam a 12,97% das despesas no triênio 2013-2015 e a TRS a mais de 5% das despesas do SUS com atenção à saúde de média e alta complexidade. Tais gastos elevados determinam grandes preocupações quanto à manutenção futura do tratamento da DRC estágio 5 no Brasil e em países em condições de desenvolvimento semelhantes ou piores.

## INTRODUCTION

Since the enactment of the Federal Constitution, in 1988, the Unified Healthcare System (SUS) was created, making access to healthcare free for the entire Brazilian population. Its final deployment took place in 1990, by Law nº 8,080, through which the healthcare system in Brazil was unified, with decentralized management; therefore, it was no longer managed exclusively by the Union, but also by States and Municipalities.^1^

With the objective of providing information for the democratization of healthcare and the improvement of its management, the DATASUS was created in 2011, the SUS Department of Information Technology, essential for the decentralization of healthcare activities and the feasibility of social control over the use of the available resources.^2^

The DATASUS website provides access to a variety of healthcare-related information available in the SUS Outpatient Information System (SIA/SUS) and in the SUS Hospital Information System (SIH/SUS).

SUS plays an important role in the care of patients with chronic kidney disease (CKD), and it is currently responsible for 90% of the treatment of patients undergoing renal replacement therapy (RRT), which includes dialysis (hemodialysis and peritoneal dialysis) and renal transplantation.

We performed the present study in order to better measure what CKD expenditures represent in our country. It is known that, at all stages, and especially in the terminal stages, CKD represents a considerable burden on the healthcare system, so it is necessary to understand it in a more comprehensive way, to properly define the public policies to be adopted in this area.

## MATERIAL AND METHODS

This is a descriptive study that used information from the Ministry of Health Data System (DATASUS) (www.datasus.saude.gov.br), through access to information in the TABNET, HealthCare in the groups: Hospital Production, SIH/SUS and SIA/SUS and SIGTAP, as follows: hospital production (expenses and hospitalizations related to kidney transplantation and simultaneous pancreas and kidney), SIH / SUS (expenses and hospitalizations related to CKD and diseases associated with CKD) and SIA/SUS (expenses for examinations for identification, follow-up and treatment of CKD, dialysis and procedures) and SIGTAP (expenses with healthcare professionals' fees and examinations used in different stages of CKD).

The values paid by the SUS during the 2013-2015 triennium were evaluated as a basis for estimating annual expenses with CKD treatments in Brazil and hospitalizations for CKD and CKD-related diseases, as well as examinations related to its diagnosis and treatment. The examinations included in this study were based on the Clinical Guidelines for the care of patients with CKD in the SUS at different stages of the disease. We categorize the treatment of renal disease in therapies administered during hospitalization for diagnosis and follow-up, dialysis and renal transplantation.

The following are among the main causes of CKD: *diabetes mellitus* (DM), systemic arterial hypertension (SAH) and cardiovascular diseases, acute myocardial infarction (AMI) and related conditions, as well as cerebrovascular accidents (Stroke) and related conditions.

CKD is currently defined as the presence of renal damage and/or reduction of the glomerular filtration rate (GFR less than 60 mL/min/1.73m^2^ body surface area) for three months or more, regardless of the cause.^3^ The current definition encompasses patients without glomerular filtration deficits, but we will refer in this article to "renal failure", considering how the patients' diagnoses were actually recorded in the DATASUS in the study period, not the current definition.

The term "procedures" used in Table 1 of the results refers to: 1) the training of patients undergoing peritoneal dialysis - APD/CAPD (9 days); 2) the implantation of catheters: catheter for subclavian of double lumen for hemodialysis; long-term catheter for hemodialysis; dual lumen catheter implant for hemodialysis; long-term catheter implantation for hemodialysis; 3) the manufacture of arteriovenous fistula for hemodialysis; 4) maintenance and home monitoring of patients submitted to APD/CAPD; 5) the manufacture of arteriovenous fistula with polytetrafluoroethylene (PTFE) grafting; 6) the creation of arteriovenous fistula with autologous graft; 7) the Tenckhoff catheter implant or similar to APD/CAPD; 8) Tenckhoff catheter implant or similar for IPD; 9) intervention in arteriovenous fistula; 10) arteriovenous fistula ligation; 11) withdrawal of Tenckhoff/long-term similar catheter.

**Table 1:** Expenditures (R$) and number of hemodialysis and peritoneal dialysis sessions and their procedures in the 2013-2015 triennium

		2013	2014	2015
IPD-1	N	508	478	263
	R$	61,843.92	58,191.72	32,017.62
IPD-2	N	4.443	3.039	2.668
	R$	539,868.93	369,268.89	324,188.68
Hemo-1	N	167.912	168.637	181.771
	R$	29,855,413.81	30,191,082.11	32,542,462.13
Hemo-3	N	12.295.381	12.809.370	12.876.765
	R$	2,184,429,080.70	2,293,261,511.10	2,305,330,675.53
Hemo HIV	N	96.743	104.476	446.295
Hep 3	R$	25,482,396.59	27,728,975.16	118,451,155.95
Hemo HIV	N	1.587	1.741	6.025
HepExcep	R$	418,349.03	462,078.81	1,599,095.25
HemoPed	N		21.605	24.409
	R$		7,645,577.40	8,637,856.92
Proced	N	246.548	254.096	266.378
	R$	32,962,130.65	69,473,964.41	72,983,181.98
Total	N	12.813.122	13.363.442	13.804.574
	R$	2,273,749,083.63	2,429,190,649.60	2,539,900,634.06

IPD-1: Intermittent peritoneal dialysis - IPD (maximum 1 session per week); IPD-2: Intermittent peritoneal dialysis - IPD (maximum 2 sessions per week); Hemo-1: hemodialysis (maximum 1 session per week - exception); Hemo-3: hemodialysis (maximum 3 sessions per week); Hemo HIV Hep 3: hemodialysis in patient with positive serology for HIV, e/or hepatitis B, and/or hepatitis C (maximum 3 sessions); Hemo HIV HepExcep: hemodialysis in patients with positive serology for HIV, and/or hepatitis B, and/or hepatitis C (exceptional); HemoPed: pediatric hemodialysis (maximum 4 sessions per week); Proced: procedures.

The statistical analysis of all the information collected in this study was made in a descriptive way, by the calculation of some summary measures, such as mean, standard deviation and relative frequency (percentage).

## RESULTS

Regarding the expenses with hemodialysis, peritoneal dialysis and related procedures, a decrease in the number of cases and in the expenditures with intermittent peritoneal dialysis (IPD), IPD-1 (Table 1) was observed during the triennium 2013-2015 (Table 1). 48.23%) and IPD-2 (39.95%); all other procedures had an increase. Increases with procedures and expenses with hemodialysis for treatment of patients with HIV and/or hepatitis B and/or C (Hemo HIV Hep3), which were 361.32% and 364.84%, and Hemo HIV Hep in exceptional situation (Excep), of 279.65% and 282.24%, respectively.

Standard hemodialysis with three weekly sessions corresponded to 95.96% of the procedures and 96.07% of the expenditures.

Data on hospitalization costs for all causes in Brazil for CKD and for CKD-associated diseases can be seen in Tables 2 and 3.

**Table 2:** Total number of hospitalizations (n) and expenditures (R$) for all causes and kidney diseases in Brazil in the 2013-2015 triennium

Diseases/Year		2013	2014	2015
All causes	N	11,197,160	11,320,287	11,372,044
	R$	12,698,359,917.70	13,370,407,625.66	13,785,610,945.46
ANS/RPGN	N	5,714	5,712	5,395
	R$	2,738,641.26	2,897,907.90	2,741,600.02
Other glomerular diseases	N	12,779	12,004	11,203
	R$	6,912,120.44	6,898,722.23	7,154,407.01
Tubulointerstitial diseases	N	92,629	90,069	86,450
	R$	34,943,288.81	36,530,932.07	36,491,157.79
CKF	N	95,186	98,220	102,110
	R$	305,589,824.67	343,252,964.84	357,376,199.04

ANS, acute nephritic syndrome; RPGN, rapidly progressive glomerulonephritis; CKF, chronic kidney failure.

**Table 3:** Total number of hospitalizations (n) and expenditures (R$) for all causes and for systemic arterial hypertension (SAH), cardiovascular and cerebrovascular diseases, and diabetes (DM) in Brazil in the 2013-2015 triennium

Diseases/Year		2013	2014	2015
All causes	N	11,197,160	11,320,287	11,372,044
	R$	12,698,359,917.70	13,370,407,625.66	13,785,610,945.46
Primary SAH	N	79,256	75,419	67,889
	R$	26,415,567.89	26,720,485.44	24,987,013.86
Other hypertensive diseases	N	26,924	26,803	24,940
	R$	15,531,169.47	13,604,391.52	14,035,285.21
Acute myocardial infarction	N	86,559	94,399	101,208
	R$	286,910,053.12	332,383,877.36	365,200,613.59
Other ischemic cardiac diseases	N	156,636	159,435	152,869
	R$	689,163,626.60	697,415,603.15	680,017,034.99
Ischemic or hemorrhagic cerebrovascular accident	N	133,822	141,909	145,970
	R$	156,146,579.87	172,055,725.92	181,525,244.48
Transitory ischemic accident and correlated syndromes	N	25,968	21,119	21,297
	R$	26,661,498.35	21,684,582.62	22,598,156.11
DM	N	140,873	139,819	138,217
	R$	88,401,913.93	89,667,747.68	92,230,156.60

Regarding hospitalization expenditures for kidney transplants performed in Brazil via SUS, during the triennium 2013-2015, most hospitalizations were for grafts from deceased donors, which corresponded to 76% of admissions and 80% of hospitalization expenditures (Table 4).

**Table 4:** Expenditures (R$) and hospitalizations (n) per kidney transplant (organ from deceased donor and live donor) and pancreas and kidney simultaneously in the 2013-2015 triennium

Transplant type		2013	2014	2015
Deceased donor	N	3,542	3,788	3,895
	R$	153,545,427.92	164,177,446.27	163,969,684.04
Live donor	N	1,129	1,145	963
	R$	35,615,018.24	36,256,637.72	30,288,077.57
Pancreas and kidney simultaneously	N	112	102	95
	R$	6,699,880.23	6,138,768.22	5,636,698.91
Total	N	4,783	5,035	4,953
	R$	195,860,326.39	206,572,852.21	199,894,460.52

Regarding the expenses with examinations for identification and treatment of CKD in the 2013-2015 triennium, there was an increase of 11.94% in the number of exams and 10.95% in the expenses. Both the number of 24-hour proteinuria tests and its related expenses increased by 32.59%. Renal biopsies had a 16.95% decrease in the number of procedures and expenditures, as can be seen in Table 5. Type I urine represented 35.53% of the exams and 47.51% of the expenditures, followed by serum creatinine (29.72% and 19.87%) and urea (25.55% and 17.08%), respectively.

**Table 5:** Expenditures (R$) and number of tests (n) used to identify, follow up and treat CKD in the 2013- 2015 triennium

Tests		2013	2014	2015
Serum creatinine	N	27,410,523	29,909,711	31,251,897
	R$	50,738,541.77	55,361,348.42	57,892,010.54
Creatinine Clearance	N	449,198	471,149	510,449
	R$	1,578,860.76	1,658,999.87	1,801,647.28
Serum urea	N	23,363,238	25,779,038	27,003,424
	R$	43,237,008.67	47,701,711.87	49,998,226.18
Type I urine	N	33,568,889	36,239,803	36,092,145
	R$	124,249,621.00	134,172,385.32	133,686,69.19
24h proteinuria	N	689,648	680,983	914,416
	R$	1,422,496.24	1,417,656.42	1,886,073.27
Urine culture	N	6,564,160	7,056,230	7,308,019
	R$	36,965,343.23	39,775,304.76	41,226,209.85
Kidney biopsy	N	838	856	696
	R$	38,707.22	40,412.45	32,148.24
Urinary tract and kidney ultrasound	N	869,384	946,936	928,680
	R$	38,707.22	40,412.45	32,148.24
Establishing the glomerular filtration rate by nuclear medicine tests	N	1,702	1,746	1,696
	R$	107,916.54	111,235.59	108,232.64
Total	N	92,917,580	101,086,452	104,011,422
	R$	258,377,202.65	280,279,467.15	286,663,395.43

## DISCUSSION

Considering the increase in CKD prevalence in the present day, one of the reasons for greater concern is that it is not due to the increase in the number of inherited renal diseases; but rather, systemic diseases that secondarily injure the kidneys, such as atherosclerosis.5, determine it.

Another factor considered relevant to the increase in terminal stage CKD in the world, besides the exponential growth of type 2 diabetes, is the aging of the population in developed countries. The incidence of the disease in the elderly (here considered individuals over 65 years of age) in the UK is above 350 pmh^6^ and in the United States it is 1200 pmh.^7^

The prevalence of CKD in the community has been largely underestimated in the past; but recent research and population studies have revealed the prevalence of CKD in the general population. In the United States, approximately 11% of the population is estimated to have CKD at some stage of evolution, according to the results of the Third National Health and Nutrition Examination Survey.^8^ About 73% of these individuals have a glomerular filtration rate of less than 60 mL/min.^9^ Even more serious is that most of these individuals fail to develop terminal CKD because they die of cardiovascular complications before progressing to the terminal stage of CKD.

In fact, it is known that patients with CKD, when compared to the general population, have a higher prevalence of cardiovascular diseases, including coronary, cerebrovascular, peripheral vascular and heart failure. In addition, the development of cardiovascular disease has been shown to be early in the course of CKD, representing the leading cause of death in the early stages of CKD^10^, or at any stage, for patients in both conservative and renal replacement therapy,^11^ which is attributed to the high prevalence of classic CVD risk factors in patients with CKD, which are associated with risk factors related to CKD itself, and which worsen as the glomerular filtration rate decreases.^12^

The prevalence of CKD has been increasing in most countries.^13^ In the present study, it was possible to see a certain trend of growth in hospitalizations and expenditures, considering all the causes of diseases occurring in Brazil during 2013 to 2015, among which there were hospitalizations for renal failure. On the other hand, hospitalizations for renal diseases "without renal failure" went down.

During the triennium, acute myocardial infarction and unspecified (hemorrhagic or ischemic) stroke had an increase in hospitalizations and expenditures, and DM only increased expenditures. The other morbidities associated with CKD presented a decrease in hospital admissions and expenses. In Brazil, cardiovascular diseases have been known to increase, which are the cause of hospitalization, morbidity and mortality, a profile that is closer to that of the industrialized countries, and not to the underdeveloped ones, in which infectious-contagious diseases predominate. The pattern observed in our study reflects precisely the change that a country like ours, in development, is suffering because of the significant increase in the number of patients with chronic diseases, especially cardiovascular disease and DM. It is worth noting that CKD has appeared more recently as a major public health concern, as already commented.

In the current study, kidney diseases (chronic renal failure, glomerular and tubulointerstitial diseases), and some of the major associated diseases (DM, SAH and other hypertensive diseases, stroke and related diseases) accounted for 7.61% of hospital admissions and 12.97% of total hospital admissions and expenses in Brazil, considering all causes. These numbers account for a large percentage of national healthcare expenditures and are only likely to increase, even if only one-offs are taken into account, such as changing the country's development profile (cited above) and population aging.^14^ It draws attention for being an expressive expense for only four diseases. However, it should be emphasized that chronic diseases are important because of the frequency that people currently develop them. It should also be borne in mind that part of the patients hospitalized due to DM, primary hypertension and other hypertensive diseases, AMI and related diseases, stroke and related diseases, among others, may also have CKD at different stages of evolution, but this diagnosis was not described as the one being responsible for the hospitalization.

Regarding renal replacement therapies, we observed that kidney transplantation with a living donor organ and simultaneous transplantation of pancreas and kidney had a decrease in hospitalizations and expenditures over the 2013-2015 triennium. On the other hand, kidney transplantation with a deceased donor organ showed a continuous increase in both instances. This finding is not surprising; the tendency to increase transplants using deceased donors has been outlining for some years, and it is currently a reality in our country. Organ donation has been encouraged in Brazil through campaigns that stimulate and encourage persons to reveal to family members their desire to be an organ donor. In fact, according to the Brazilian Registry of Transplantation (ABTO), 78.9% of kidney transplants in Brazil in 2015 were performed with organs from deceased donors; thus, only a small portion depended on living donors. The ABTO data also showed that, in the last ten years, kidney transplants with deceased organ donors had an increase of 170.95%, with a 33% decrease in kidney transplantation with live donor, and simultaneous 14.91% pancreas and kidney transplantation.^15^

Intermittent peritoneal dialysis (one and two sessions per week) experienced a decrease in procedures and expenditures; the other dialysis procedures increased, with emphasis on the growth of procedures and expenses with hemodialysis in patients with positive serology for HIV and/or hepatitis B and/or hepatitis C (maximum 3 sessions), and hemodialysis in patients with positive serology for HIV, and/or hepatitis B, and/or hepatitis C (exceptionality). The reason for increased spending on hemodialysis sessions for HIV-positive patients and/or those with viral hepatitis was unclear, but there are some possibilities, such as the increase in the number of patients with these conditions who survived the initial phase of the disease due to the current therapeutic resources and have managed to reach the dialysis, or even the increase in the number of cases of these diseases, or only in the number of diagnoses, previously not performed, which is more likely.

It is also worth noting that in Brazil, in July 2014, 91.4% of patients on chronic dialysis were treated by hemodialysis, and 8.6% by peritoneal dialysis, and automated peritoneal dialysis was the predominant modality. The percentage of patients undergoing maintenance hemodialysis has remained stable, and there is a trend towards an overall increase in the number of dialysis patients, incidence rates and treatment prevalence, particularly considering the last four years. It is important to say that the payment for these procedures in Brazil is done predominantly by the SUS.^5^

A study by Menezes et al.^15^ explored the scenario of hemodialysis treatment paid by the SUS in Brazil, emphasizing that almost half of the country's hemodialysis expenditures were concentrated in São Paulo, Rio de Janeiro and Minas Gerais. This is another very important aspect, the inequality of treatment performed, which is independent of the population of each region, also documented by Sesso et al.^5^

In order to have an idea of the extent of RRT expenditures in Brazil compared to those for various diseases, it is worth recalling some spending figures for the year 2015. Thus, for all-cause hospitalizations we spent 13.8 billion Reals and, according to our survey, more than 2 billion with RRT (about 200 million with renal transplantation and 2 billion with dialysis), not included here there are approximately 357 million spent on patients with kidney failure. As a comparison, hospitalizations for acute myocardial infarction and related diseases accounted for about 1 billion reals in the same year, or less than half of that for RRT in Brazil.

Considering what was disclosed for the total SUS spending in 2015, with the medium and high complexity healthcare procedures in the Brazilian population, that is, 40 billion reals, it is worth noting that the expenses with RRT (i.e. only with CKD treatment in stage 5) accounted for more than 2 billion Reals, and 2 billion correspond to 5% of the SUS costs with medium and high complexity treatments, consumed with part of the management of a single disease, the incidence of which is increasing.

It must be said that a study based primarily on the number and expenditures on hospitalizations motivated by selected diagnoses, such as ours, has its limitations. The information available is true, but it is known that certain data or diagnoses may be lacking. Still, it is generally considered as having good reliability. In fact, several cost studies have been carried out using these databases, in order to evaluate the economic impact of some causes of hospital admissions in our country.^16^

In Brazil, hospital statistics are better systematized than outpatient statistics,^17^ therefore hospitalization expenditure surveys are used as a way of measuring healthcare costs, as we did in the present study.

When considering current trends in the prevalence of CKD, RRT will probably be difficult to maintain in a time not too distant, even for industrialized countries,^18^ hence the importance of having expenditures studies in this area, seeking to alert about the problem and the need to look for alternatives, such as early prevention, diagnosis and treatment of CKD.
